# Vulcanite Ideas

**Published:** 1860-07

**Authors:** A. Packham

**Affiliations:** Whistesville, Harries Co., Ga.


### VULCANITE IDEAS.

As the Vulcanite and Amber base continues to occupy the attention of the dental profession, almost to the exclusion of every other subject, a few *new* ideas relative to the manipulating of the same, will, I hope, be received with favor; so I'll to the point at once.

The base, which insinuates itself between the teeth, no matter with what accuracy you grind them, will invariably show itself, after the piece is vulcanized, without adding to its artistic appearance; here is an idea which will effectually prevent it: apply Pearson's Osteoplastic to the joints, upon the *final* arrangement of the teeth. I have tried Plaster, Soluble Glass, Diamond Cement, and *Spalding's Glue*, but the Osteplastic is superior to them all.

Another idea!! After you have arranged your teeth to the temporary plate, do not put melted wax around, on, or between the teeth, but take a strip of tin foil and place that over the pins, then put wax over the tin foil; by so doing you save over an hour's labor, for you can (after you have invested the job in the flask, previous to packing,) take hold of the foil, and pull off the wax from the teeth at once; besides having the pins perfectly clean, which is absolutely necessary for their perfect union with the base.

Another idea!! Another hour's labor will be saved if you have the temporary plate so thin that it will plainly indicate the form of the ruga and chamber when pressed on the model; by filling the depressions of these with wax, you will have it sufficiently thin, after being vulcanized without one third the usual trouble of dressing down.

Another idea!! Before you pack cut a circle around the model in addition to the usual channels cut, also file a few notches into the rim of the flask itself, running the channels in a line with them, then the superabundance of material will exude out, and allow the two sections to meet, thereby preventing what has heretofore been a great annoyance—the perfect antagonism of the teeth.

Another idea!! Heat your flasks and base, inside of your vulcanizer (empty) instead of on a heated brick or lamp.

*Au revoir.* A. PACKHAM.

*Whitesville, Harris Co., Ga.*, May 1, 1860.

